# A clinical pathway for community-acquired pneumonia: an observational cohort study

**DOI:** 10.1186/1471-2334-11-188

**Published:** 2011-07-06

**Authors:** Christopher R Frei, Allison M Bell, Kristi A Traugott, Terry C Jaso, Kelly R Daniels, Eric M Mortensen, Marcos I Restrepo, Christine U Oramasionwu, Andres D Ruiz, William R Mylchreest, Vanja Sikirica, Monika R Raut, Alan Fisher, Jeff R Schein

**Affiliations:** 1 College of Pharmacy, The University of Texas at Austin, 1 University Station A1900, Austin TX, 78712, USA; 2 Pharmacotherapy Education and Research Center, School of Medicine, The University of Texas Health Science Center at San Antonio, 7703 Floyd Curl Drive, Mail Code 6220, San Antonio, TX, 78229-3900, USA; 3 Department of Pharmacy, South Texas Veterans Healthcare System, ALMD/UTHSCSA, 7400 Merton Minter Boulevard, San Antonio, TX, 78229, USA; 4 Seton Family of Hospitals, P.O. Box 201233, Austin, TX, 78720-1233, USA; 5 Oakdell Pharmacy, Inc., 7220 Louis Pasteur Drive, Suite 176, San Antonio, TX, 78229, USA; 6 Department of Medicine, The University of Texas Health Science Center at San Antonio, 7703 Floyd Curl Drive, San Antonio, TX, 78229-3900, USA; 7 VERDICT, South Texas Veterans Healthcare System, ALMD/UTHSCSA, Ambulatory Care (11C6), 7400 Merton Minter Boulevard, San Antonio, TX, 78229, USA; 8 Ortho-McNeil Janssen Scientific Affairs, LLC, 1000 Route 202, P.O. Box 300, Raritan, NJ, 08869, USA

## Abstract

**Background:**

Six hospitals instituted a voluntary, system-wide, pathway for community acquired pneumonia (CAP). We proposed this study to determine the impact of pathway antibiotics on patient survival, hospital length of stay (LOS), and total hospital cost.

**Methods:**

Data were collected for adults from six U.S. hospitals with a principal CAP discharge diagnosis code, a chest infiltrate, and medical notes indicative of CAP from 2005-2007. Pathway and non-pathway cohorts were assigned according to antibiotics received within 48 hours of admission. Pathway antibiotics included levofloxacin 750 mg monotherapy or ceftriaxone 1000 mg plus azithromycin 500 mg daily. Multivariable regression models assessed 90-day mortality, hospital LOS, total hospital cost, and total pharmacy cost.

**Results:**

Overall, 792 patients met study criteria. Of these, 505 (64%) received pathway antibiotics and 287 (36%) received non-pathway antibiotics. Adjusted means and p-values were derived from Least Squares regression models that included Pneumonia Severity Index risk class, patient age, heart failure, chronic obstructive pulmonary disease, and admitting hospital as covariates. After adjustment, patients who received pathway antibiotics experienced lower adjusted 90-day mortality (*p *= 0.02), shorter mean hospital LOS (3.9 vs. 5.0 days, *p *< 0.01), lower mean hospital costs ($2,485 vs. $3,281, *p *= 0.02), and similar mean pharmacy costs ($356 vs. $442, *p *= 0.11).

**Conclusions:**

Pathway antibiotics were associated with improved patient survival, hospital LOS, and total hospital cost for patients admitted to the hospital with CAP.

## Background

The Infectious Diseases Society of America (IDSA), in conjunction with the American Thoracic Society (ATS), has published guidelines for the empiric treatment of CAP in adults [1]. Regarding ward patients, these guidelines advocate fluoroquinolone monotherapy or combination therapy with a beta-lactam plus a macrolide [1]. The 2007 guidelines were the first to specify a dose for one of the fluoroquinolones (i.e., levofloxacin 750 mg daily) [1]. Though not explicitly stated in the guidelines, it is known from the pharmacokinetic-pharmacodynamic (PK-PD) literature that higher daily fluoroquinolone doses allow for greater antibiotic lung penetration [2]. Theoretically, the use of PK-PD based dosing should result in additional patient benefits beyond those seen with previous versions of the guidelines which did not specify PK-PD based dosing [3]. This study aimed to test this theory by comparing health and economic outcomes for CAP patients treated according to a clinical pathway to those patients not treated according to the pathway.

## Methods

The study setting was a six-hospital health-system in Austin, TX and its surrounding communities. In 2004, these hospitals began to participate in the Center for Medicare and Medicaid Services (CMS) Pneumonia Core Measures. Staff and quality control personnel discovered suboptimal compliance with guideline-endorsed initial antibiotics. Therefore, a multi-disciplinary, disease-state management team designed and implemented a voluntary, system-wide clinical pathway for the management of hospitalized CAP patients in 2005. The pathway included standard orders for antibiotic selection, including levofloxacin 750 mg daily or ceftriaxone 1000 mg plus azithromycin 500 mg daily. The implementation of this pathway led to antibiotic streamlining and set the stage for this comparative-effectiveness evaluation.

This study was approved by institutional review boards (IRB) at two universities (The University of Texas at Austin and The University of Texas Health Science Center at San Antonio) and one central IRB representing all six hospitals (Seton Family of Hospitals, Brackenridge Hospital). Data were extracted from medical charts for adults (age 18 years or older) with a principal discharge diagnosis of pneumonia (ICD-9-CM codes 481-484 and 486) between January 2005 and December 2007. To minimize the impact of coding errors, only patients with a clinical diagnosis of pneumonia (medical progress notes) and documentation of a chest infiltrate (radiology notes) were included. Patients were excluded if they had risk factors for healthcare-associated pneumonia (HCAP), which included the following: admission from a nursing home or other long-term care facility, transfer from another acute care hospital, dialysis, hospital admission in the last 90 days, or indwelling catheter or percutaneous medical device. Patients were also excluded if they were immunocompromised (e.g., history of HIV/AIDS, transplant, or current chemotherapy), experienced an abbreviated stay (e.g., discharged to another acute care hospital, left against medical advice, were hospitalized for only 1 day, or died on day 1 of admission), or were admitted directly to the intensive care unit (ICU). Finally, patients were excluded if they had renal disease or an estimated creatinine clearance (CrCl) less than 50 mL/min. CrCl was estimated using the Cockcroft Gault equation. Ideal body weight was used for patients with a body mass index more than 30 kg/m^2^; whereas, actual body weight was used for patients with a body mass index of 30 kg/m^2 ^or less.

Data collection included information regarding admission year, facility, admitting service, patient demographics, past medical history, past social history, antibiotics prescribed during hospitalization and at discharge, daily vital signs, culture results, hospital LOS, total pharmacy costs, and total hospital costs. Severity of illness was determined according to the Pneumonia Severity Index (PSI), a well-validated rule that includes patient history, comorbidities, presenting vital signs and symptoms, and baseline laboratory and diagnostic test results [4]. Ninety-day mortality was determined by linking to the United States Social Security Death Index. This index enabled mortality tracking post-discharge.

### Data and Statistical Analyses

The primary endpoint was 90-day mortality. Secondary endpoints included hospital LOS, total hospital costs, and total pharmacy costs. LOS was determined by the following equation: LOS = (discharge date - admission date) + 1 day. Patients were divided into two subgroups on the basis of antibiotics received during the first two days of hospitalization: (1) pathway antibiotics or (2) non-pathway antibiotics. Pathway antibiotics included levofloxacin 750 mg IV or PO daily or ceftriaxone 1000 mg IV plus azithromycin 500 mg IV or PO daily. Patients who received these antibiotics at higher doses were also included in the pathway group. Patients who received these antibiotics at lower doses, or alternative antibiotics, were stratified to the non-pathway group. The following characteristics were compared between the two groups: age, sex, nine key comorbidities, substance abuse, severity of illness, microbial etiology, 90-day mortality, hospital LOS, and cost.

JMP 7.0^® ^(SAS Corp, Cary, NC) was used for all statistical analyses. Statistical comparisons with p-values < 0.05 were considered statistically significant. Chi-square, Fisher's Exact, and chi-square test for ordinal data were used to compare discrete baseline variables, whereas the Student's t-test was used to compare continuous baseline variables. A multivariable logistic regression model was created to assess the impact of the pathway antibiotics on 90-day mortality. The other three outcomes (hospital LOS, total hospital cost, and total pharmacy cost) were assessed in multivariable Least Squares regression models. PSI risk class, age, heart failure, renal disease, chronic obstructive pulmonary disease (COPD), and admitting hospital were included as covariates in each of these models.

## Results

A total of 792 patients from six hospitals met study criteria. Of these, 505 (64%) received pathway antibiotics and 287 (36%) received other therapies. The most common pathway therapies included: levofloxacin 750 mg daily (n = 336, 67%) and combination therapy with ceftriaxone 1000 mg and azithromycin 500 mg, daily (n = 169, 33%). Common non-pathway therapies included levofloxacin 500 mg daily (n = 188, 66%) and levofloxacin 250 mg daily (n = 20, 7%). Pathway and non-pathway groups were similar with respect to sex, race, substance abuse history, and admitting service; however, non-pathway patients were older, with more COPD (Table 1).

**Table 1:** Patient demographics by initial antibiotic regimen *, †

Characteristic	Pathway	Non-Pathway	*p*-value
	**(n = 505)**	**(n = 287)**	

Age (yrs), median (IQR)	58 (45-71)	68 (51-81)	***<0.01***

Sex			

Male	46%	40%	0.1

Female	54%	60%	--

Race			

White	68%	71%	0.5

Hispanic	17%	14%	--

Black	11%	10%	--

Other	4%	5%	--

Comorbidities			

Neoplastic disease	10%	13%	0.2

Liver disease	6%	8%	0.2

Heart failure	10%	14%	0.06

Cerebrovascular disease	5%	7%	0.4

Diabetes mellitus	24%	21%	0.5

COPD	19%	26%	***0.02***

Asthma	15%	16%	0.9


Depression	9%	10%	0.5


Social history			

Tobacco (smoker)	28%	30%	0.5

Alcohol	16%	15%	0.6

Intravenous drug abuse	3%	3%	0.9

Admitting Service			

Medicine	92%	92%	0.9

Other	8%	8%	--

* Pathway = levofloxacin 750 mg intravenous or oral daily OR ceftriaxone 1000 mg intravenous plus azithromycin 500 mg intravenous or oral daily OR either of these antibiotic regimens at higher doses; Non-pathway = these antibiotics at lower doses OR alternative antibiotics; COPD = chronic obstructive pulmonary disease.

† The following statistical tests were used: Chi-square (sex, race, individual comorbidities, social history, and admitting service) and Student's t-test (age).

Pathway and non-pathway groups differed with respect to PSI risk class (*p *< 0.01), but were well-balanced with respect to bacterial etiology (p = 0.2). The proportions of pathway and non-pathway patients in each of the five PSI risk classes were as follows: I (15% vs. 12%), II (44% vs. 34%), III (24% vs. 26%), IV (16% vs. 26%), and V (1% vs. 2%). Pathway and non-pathway patients were frequently not cultured (42% vs. 39%) or were culture-negative (44% vs. 51%). When a bacterial pathogen was identified among pathway and non-pathway patients, it was most frequently *Streptococcus pneumoniae *(8% vs. 5%), *Haemophilus influenzae *(2% vs. 1%), *Staphylococcus aureus *(2% vs. 2%), *Moraxella catarrhalis *(1% vs. 0%), or other (1% vs. 2%).

Crude 90-day mortality was significantly lower in patients who received pathway antibiotics versus non-pathway antibiotics (1.4% vs. 4.5%, *p *< 0.01). This finding was also statistically significant in an adjusted logistic regression model that included PSI risk class, patient age, heart failure, COPD, and admitting hospital as covariates (*p *= 0.02).

**Figure 1:**
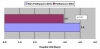
Adjusted least squares mean hospital length of stay by initial antibiotic regimen*. * The adjusted mean and *p-*value were derived from a least squares regression model that included PSI risk class, patient age, heart failure, chronic obstructive pulmonary disease, and admitting hospital as covariates.

**Figure 2:**
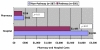
Adjusted least squares mean pharmacy and hospital costs by initial antibiotic regimen*. * Adjusted means and *p-*values were derived from least squares regression models that included PSI risk class, patient age, heart failure, chronic obstructive pulmonary disease, and admitting hospital as covariates.

Mean hospital LOS (4.9 vs. 6.0 days, *p *< 0.01) and total hospital costs ($3,184 vs. $4,168, *p *< 0.01) were significantly less for patients treated with pathway vs. non-pathway therapies; however, total pharmacy costs ($528 vs. $611, *p *= 0.12) were similar. Adjusted means and p-values were derived from Least Squares regression models that included PSI risk class, patient age, heart failure, COPD, and admitting hospital as covariates. Adjusted mean hospital LOS was significantly shorter for patients treated with pathway vs. non-pathway therapies (3.9 vs. 5.0 days, *p *< 0.01) (Figure 1). Adjusted hospital costs were lower with pathway antibiotics ($2,485 vs. $3,281, *p *= 0.02) and adjusted total pharmacy costs were similar ($356 vs. $442, *p *= 0.11) (Figure 2). These results demonstrate that, on average, the pathway resulted in one day shorter time to hospital discharge and a reduced total hospital cost of $796 per patient. This represents a potential difference of 9 lives saved, 287 hospital days saved, and $228,452 saved if all of the 287 patients in the non-pathway group had been treated with pathway antibiotics.

## Discussion

This study demonstrates that pathway antibiotics are associated with improved survival, shorter hospital LOS, and reduced total hospital cost. This pathway is unique in that it uses PK-PD dosing. In so doing, this study goes beyond traditional guideline-concordant studies by examining antibiotic dose and regimen, rather than antibiotic choice alone. Our chief finding was a reduction in mortality for patients who received pathway antibiotics. We also observed a decrease in hospital LOS and total hospital costs in CAP patients treated with pathway antibiotics, despite similar total pharmacy costs. Reducing LOS may have other benefits including reduced invasive catheter use, decreased risk of nosocomial diseases, and enhanced patient quality of life [5,6].

Fluoroquinolone monotherapy has been a first-line recommendation for the empiric treatment of hospital ward patients in several versions of the CAP guidelines [1,3]; however, the 2007 IDSA/ATS guidelines were the first to specify a levofloxacin dose of 750 mg daily [1]. The reason was not explicitly stated; however, the rationale for this recommendation can be surmised from recent PK-PD and clinical literature touting the benefits of levofloxacin 750 mg daily [2]. Fluoroquinolones are considered to be concentration-dependent antibiotics, meaning higher drug concentrations result in greater rates and extent of microbial killing [7,8]. Favorable outcomes have been associated with C_max_/MIC, though the pharmacodynamic parameter most commonly correlated with antibiotic efficacy is the 24-hour area under the curve/MIC (AUC_24_/MIC) ratio [9,10]. For Gram-positive infections, the optimal AUC_24_/MIC is 30 to 40 [11].

The approved dosing regimens for levofloxacin in CAP are 500 mg daily for 7-14 days or 750 mg daily for 5 days. An *in vitro *study by Lister examined the pharmacodynamics of levofloxacin 500 mg and 750 mg against wild type *S. pneumoniae *strains and ciprofloxacin resistant strains [12]. In organisms that exhibited a higher MIC_50_, the 750 mg dose eradicated the infective organism more effectively than the 500 mg dose [12]. Dunbar *et al*. performed a multicenter, randomized, double-blind investigation comparing levofloxacin 750 mg daily for 5 days vs. levofloxacin 500 mg daily for 10 days in patients with mild to severe CAP [13]. Levofloxacin 750 mg daily demonstrated comparable efficacy to 500 mg daily in clinical success rates, with similar results seen in a subgroup analysis of PSI class III/IV patients [14], as well as in elderly patients [15]. However, in comparison to levofloxacin 500 mg, levofloxacin 750 mg was associated with a significantly shorter time to fever reduction, faster resolution of purulent sputum and a trend towards more rapid IV to PO conversion with no significant increases in adverse events [13,14,16,17]. Few studies have examined different dosage regimens of moxifloxacin, though one demonstrated increased efficacy for 400 mg vs. 200 mg [18].

Beta-lactams, in combination with macrolides, have also been a first-line recommendation for the empiric treatment of hospital ward patients throughout several versions of the CAP guidelines. The 2007 IDSA/ATS guidelines do not specify antibiotic doses or dosing regimens for any of the beta-lactams. We know from PK-PD studies that beta-lactams demonstrate a time-dependent mechanism for bacterial killing and the pharmacodynamic parameter most closely associated with outcomes is percent time above the MIC (%T>MIC). A %T>MIC of approximately 40-50% of the dosing interval is usually considered adequate [7]. Options to maximize the PK-PD of beta-lactams include more frequent dosing, continuous infusions, and extended infusion.

Macrolides were the final class of antibiotics used in our pathway. The 2007 IDSA/ATS guidelines support the use of azithromycin in combination with a beta-lactam as first-line therapy for CAP ward patients, but the guidelines do not specify a dose for azithromycin [1]. Approved azithromycin doses for CAP include: 500 mg IV daily for 2 days, followed by 500 mg PO daily to complete a 7-10 day antibiotic course; 500 mg PO once followed by 250 mg PO daily for four more days; or a one-time dose of 2000 mg PO. The product labeling does not specify doses for either outpatients or inpatients. Most studies of azithromycin in combination with ceftriaxone for ward patients have used an initial 500 mg IV dose for at least two days followed by oral therapy, although guidelines recommend either IV or PO azithromycin when used in combination with beta-lactams [1,19-21]. Conventional macrolides are considered to be time-dependent antibiotics; however, azithromycin is thought to be unique. Azithromycin achieves extremely high, sustained concentrations in tissues and cells. Because of this, AUC_24_/MIC is the PK-PD parameter associated with improved clinical outcomes [22]. The target range of 25-30 has been correlated with improved outcomes in patients with *S. pneumoniae *CAP [23].

Our study has strengths and limitations. First, we used ICD-9-CM codes and chest radiographs to define our cohort, thereby minimizing the impact of coding errors introduced when using diagnosis codes alone. A second strength is our meticulous exclusion of patients with HCAP risk factors, which is directly responsible for the lower PSI scores in our patients compared to the scores seen in other published studies [24,25]. Not only are HCAP patients at greater risk for *S. aureus*, but they are also at increased risk for methicillin-resistant *S. aureus *(MRSA) [26]. The primary limitation of our study is the retrospective nature of the analysis, which limits the ability to detect differences in groups caused by unmeasured variables. There were significant differences in the baseline characteristics of the two populations. In the "pathway" group, the patients were younger, with less heart failure, and COPD. These factors were introduced as covariates in the multivariable regression models; however, these factors may have been related to other, unmeasured variables, and these might have influenced patient outcomes. Antibiotic timing was not collected or evaluated, but differences in antibiotic timing between the groups could also influence the results. Positive cultures were obtained for only 10% of our patients, which may limit the validity of our pathogen distributions. Finally, this study specifically compares those patients who were initially treated with pathway versus non-pathway antibiotics; therefore, other aspects of treatment, such as time to switch therapy, duration of treatment, and compliance may have unknowingly influenced outcomes.

## Conclusions

Pathway antibiotics were associated with improved patient survival, hospital LOS, and total hospital cost for patients admitted to the hospital with CAP.

## Competing interests

CRF has received research grants and/or served as a scientific consultant/advisor for the NIH, AstraZeneca, Elan, Ortho McNeil Janssen Pharmaceuticals, and Pfizer. MIR is on the speaker's bureaus of Ortho-McNeil Janssen, Johnson & Johnson, Pfizer, and BARD, Inc. He has also served on Advisory Boards for Forest, Ortho-McNeil Janssen, Johnson & Johnson, Pfizer, and BARD, Inc. VS, MRR, AF, and JRS are current or former employees of Ortho McNeil Janssen Scientific Affairs, LLC. AMB, KAT, TCJ, KRD, EMM, CUO, ADR, and WRM have nothing to disclose related to the content of this manuscript.

## Authors' contributions

CRF had full access to study data and was the primary person involved in the study design, study concepts, data collection, data analysis, data interpretation, and manuscript drafting. AMB, KAT, and KRD drafted the original introduction and discussion sections of the manuscript. TCJ and MIR were involved in the study design and manuscript editing. EMM was involved in the study design, data analysis, and manuscript editing. CUO and ADR were involved in data collection and manuscript editing. WRM helped obtain and analyze the cost data. VS, MRR, AF, and JRS were involved in manuscript review for important intellectual content. All authors read and approved the final manuscript.

## Source of Funding

Funding was provided by Ortho-McNeil Janssen Scientific Affairs, LLC. The study was designed locally and all data were collected and analyzed by the local investigators. The sponsor set no limitations on study content. CRF is supported by the United States National Institutes of Health in the form of a KL2 career development award (KL2 RR025766).

## Pre-publication history

The pre-publication history for this paper can be accessed here:

http://www.biomedcentral.com/1471-2334/11/188/prepub

## References

[B1] Mandell LA Wunderink RG Anzueto A Infectious Diseases Society of America/American Thoracic Society consensus guidelines on the management of community-acquired pneumonia in adults Clin Infect Dis 2007 44 Suppl 2 S27 72 17278083 10.1086/511159 PMC7107997

[B2] Conte JE Jr Golden JA McIver M Zurlinden E Intrapulmonary pharmacokinetics and pharmacodynamics of high-dose levofloxacin in healthy volunteer subjects Int J Antimicrob Agents 2006 28 114 21 10.1016/j.ijantimicag.2006.03.022 16837169

[B3] Mandell LA Bartlett JG Dowell SF File TM Jr Musher DM Whitney C Update of practice guidelines for the management of community-acquired pneumonia in immunocompetent adults Clin Infect Dis 2003 37 1405 33 10.1086/380488 14614663 PMC7199894

[B4] Fine MJ Auble TE Yealy DM A prediction rule to identify low-risk patients with community-acquired pneumonia N Engl J Med 1997 336 243 50 10.1056/NEJM199701233360402 8995086

[B5] Guven GS Uzun O Principles of good use of antibiotics in hospitals J Hosp Infect 2003 53 91 6 10.1053/jhin.2002.1353 12586566

[B6] Restrepo MI Frei CR Health economics of use fluoroquinolones to treat patients with community-acquired pneumonia Am J Med 2010 123 S39 46 10.1016/j.amjmed.2010.02.005 20350634

[B7] Owens RC Jr Shorr AF Rational dosing of antimicrobial agents: pharmacokinetic and pharmacodynamic strategies Am J Health Syst Pharm 2009 66 S23 30 10.2146/090087d 19502225

[B8] Craig WA Pharmacokinetic/pharmacodynamic parameters: rationale for antibacterial dosing of mice and men Clin Infect Dis 1998 26 1 10 10.1086/516284 9455502

[B9] Forrest A Nix DE Ballow CH Goss TF Birmingham MC Schentag JJ Pharmacodynamics of intravenous ciprofloxacin in seriously ill patients Antimicrob Agents Chemother 1993 37 1073 81 8517694 10.1128/aac.37.5.1073 PMC187901

[B10] Preston SL Drusano GL Berman AL Pharmacodynamics of levofloxacin: a new paradigm for early clinical trials JAMA 1998 279 125 9 10.1001/jama.279.2.125 9440662

[B11] Ambrose PG Grasela DM Grasela TH Passarell J Mayer HB Pierce PF Pharmacodynamics of fluoroquinolones against *Streptococcus pneumoniae *in patients with community-acquired respiratory tract infections Antimicrob Agents Chemother 2001 45 2793 7 10.1128/AAC.45.10.2793-2797.2001 11557471 PMC90733

[B12] Lister PD Pharmacodynamics of 750 mg and 500 mg doses of levofloxacin against ciprofloxacin-resistant strains of *Streptococcus pneumoniae* Diagn Microbiol Infect Dis 2002 44 43 9 10.1016/S0732-8893(02)00417-0 12376030

[B13] Dunbar LM Wunderink RG Habib MP High-dose, short-course levofloxacin for community-acquired pneumonia: a new treatment paradigm Clin Infect Dis 2003 37 752 60 10.1086/377539 12955634

[B14] Shorr AF Khashab MM Xiang JX Tennenberg AM Kahn JB Levofloxacin 750-mg for 5 days for the treatment of hospitalized Fine Risk Class III/IV community-acquired pneumonia patients Respir Med 2006 100 2129 36 10.1016/j.rmed.2006.03.019 16730170

[B15] Shorr AF Zadeikis N Xiang JX Tennenberg AM Wes Ely E A multicenter, randomized, double-blind, retrospective comparison of 5- and 10-day regimens of levofloxacin in a subgroup of patients aged > or = 65 years with community-acquired pneumonia Clin Ther 2005 27 1251 9 10.1016/S0149-2918(05)80214-0 16199249

[B16] File TM Jr Milkovich G Tennenberg AM Xiang JX Khashab MM Zadeikis N Clinical implications of 750 mg, 5-day levofloxacin for the treatment of community-acquired pneumonia Curr Med Res Opin 2004 20 1473 81 10.1185/030079904X2556 15383197

[B17] Khashab MM Xiang J Kahn JB Comparison of the adverse event profiles of levofloxacin 500 mg and 750 mg in clinical trials for the treatment of respiratory infections Curr Med Res Opin 2006 22 1997 2006 10.1185/030079906X132505 17022859

[B18] Hoeffken G Meyer HP Winter J Verhoef L The efficacy and safety of two oral moxifloxacin regimens compared to oral clarithromycin in the treatment of community-acquired pneumonia Respir Med 2001 95 553 64 10.1053/rmed.2001.1113 11453311

[B19] Rubio FG Cunha CA Lundgren FL Intravenous azithromycin plus ceftriaxone followed by oral azithromycin for the treatment of inpatients with community-acquired pneumonia: an open-label, non-comparative multicenter trial Braz J Infect Dis 2008 12 202 9 10.1590/S1413-86702008000300008 18833404

[B20] Tamm M Todisco T Feldman C Clinical and bacteriological outcomes in hospitalised patients with community-acquired pneumonia treated with azithromycin plus ceftriaxone, or ceftriaxone plus clarithromycin or erythromycin: a prospective, randomised, multicentre study Clin Microbiol Infect 2007 13 162 71 17328728 10.1111/j.1469-0691.2006.01633.x

[B21] Frank E Liu J Kinasewitz G A multicenter, open-label, randomized comparison of levofloxacin and azithromycin plus ceftriaxone in hospitalized adults with moderate to severe community-acquired pneumonia Clin Ther 2002 24 1292 308 10.1016/S0149-2918(02)80034-0 12240780

[B22] Van Bambeke F Tulkens PM Macrolides: pharmacokinetics and pharmacodynamics Int J Antimicrob Agents 2001 18 Suppl 1 S17 23 11574190 10.1016/s0924-8579(01)00406-x

[B23] Noreddin AM El-Khatib WF Aolie J Salem AH Zhanel GG Pharmacodynamic target attainment potential of azithromycin, clarithromycin, and telithromycin in serum and epithelial lining fluid of community-acquired pneumonia patients with penicillin-susceptible, intermediate, and resistant *Streptococcus pneumoniae* Int J Infect Dis 2009 13 483 7 10.1016/j.ijid.2008.08.016 19046911

[B24] Malone DC Shaban HM Adherence to ATS guidelines for hospitalized patients with community-acquired pneumonia Ann Pharmacother 2001 35 1180 5 11675841 10.1345/aph.10283

[B25] Reyes Calzada S Martinez Tomas R Cremades Romero MJ Martinez Moragon E Soler Cataluna JJ Menendez Villanueva R Empiric treatment in hospitalized community-acquired pneumonia. Impact on mortality, length of stay and re-admission Respir Med 2007 101 1909 15 10.1016/j.rmed.2007.04.018 17628462

[B26] Shorr AF Zilberberg MD Micek ST Kollef MH Prediction of infection due to antibiotic-resistant bacteria by select risk factors for health care-associated pneumonia Arch Intern Med 2008 168 2205 10 10.1001/archinte.168.20.2205 19001196

